# Combining self-organizing mapping and supervised affinity propagation clustering approach to investigate functional brain networks involved in motor imagery and execution with fMRI measurements

**DOI:** 10.3389/fnhum.2015.00400

**Published:** 2015-07-17

**Authors:** Jiang Zhang, Qi Liu, Huafu Chen, Zhen Yuan, Jin Huang, Lihua Deng, Fengmei Lu, Junpeng Zhang, Yuqing Wang, Mingwen Wang, Liangyin Chen

**Affiliations:** ^1^Department of Medical Information Engineering, School of Electrical Engineering and Information, Sichuan UniversityChengdu, China; ^2^School of Life Science and Technology, University of Electronic Science and Technology of ChinaChengdu, China; ^3^Bioimaging Core, Faculty of Health Sciences, University of MacauMacau, China; ^4^School of Foreign Studies, University of Electronic Science and Technology of ChinaChengdu, China; ^5^CAS Key Laboratory for Biomedical Effects of Nanomaterials and Nanosafety National Center for Nanoscience and Technology of ChinaBeijing, China; ^6^School of Mathematics, Southwest Jiaotong UniversityChengdu, China; ^7^School of Computer Science, Sichuan UniversityChengdu, China

**Keywords:** motor imagery, motor execution, functional magnetic resonance imaging, self-organizing mapping, affinity propagation clustering

## Abstract

Clustering analysis methods have been widely applied to identifying the functional brain networks of a multitask paradigm. However, the previously used clustering analysis techniques are computationally expensive and thus impractical for clinical applications. In this study a novel method, called SOM-SAPC that combines self-organizing mapping (SOM) and supervised affinity propagation clustering (SAPC), is proposed and implemented to identify the motor execution (ME) and motor imagery (MI) networks. In SOM-SAPC, SOM was first performed to process fMRI data and SAPC is further utilized for clustering the patterns of functional networks. As a result, SOM-SAPC is able to significantly reduce the computational cost for brain network analysis. Simulation and clinical tests involving ME and MI were conducted based on SOM-SAPC, and the analysis results indicated that functional brain networks were clearly identified with different response patterns and reduced computational cost. In particular, three activation clusters were clearly revealed, which include parts of the visual, ME and MI functional networks. These findings validated that SOM-SAPC is an effective and robust method to analyze the fMRI data with multitasks.

## Introduction

Motor imagery (MI) and motor execution (ME), have shown their potentials for the investigations of rehabilitation in movement disorders and brain-computer interfaces (BCIs) as well as for training athletes and musicians (Jeannerod, 1995; Porro et al., 1996; Pfurtscheller and Neuper, 2001; Malouin et al., 2004; Brouziyne and Molinaro, 2005; Kimberley et al., 2006; Lotze and Halsband, 2006; Felton et al., 2007; Chen et al., 2009). Now advances in brain imaging are undergoing a transition from mapping sites of cortical activations toward identifying the brain networks that connect these sites together into dynamic systems in time-frequency domain (Buxton et al., 2004; Ding et al., 2006; Yuan and Ye, 2013). As such, quantifications of brain networks have become a major field of interest in neuroimaging and neurointegration for the investigation of MI and ME (Ding et al., 2006; Chen et al., 2009; Wang et al., 2010; Yuan and Ye, 2013). For assessing functional connectivity in human brains involved in MI and ME, it is essential to conduct pattern clustering of complex brain networks using fMRI data.

Interestingly clustering analysis methods like self-organizing mapping (SOM) and affinity propagation clustering (APC) are able to reveal inter-subject differences in the temporal dynamics of the fMRI signals without a prior model (Goutte et al., 1999; Frey and Dueck, 2007; Liao et al., 2008; Ren et al., 2014). SOM (Kohonen, 1982, 1990, 1995; Haykin, 1999) transforms the incoming signal patterns into low dimensional discrete maps (Kohonen, 1990, 1995; Haykin, 1999) to generate time courses of a predetermined number of exemplars (Peltier et al., 2003). Meanwhile, APC utilizes a pattern cluster tool that is able to sort out exemplars from the whole data points, in which clusters of data points surrounding the specific exemplars are generated (Frey and Dueck, 2007). However, although APC is considered an efficient analysis method (Frey and Dueck, 2007; Mézard, 2007), it is very hard to use this scheme to conduct pattern clustering of complex functional networks using fMRI measurements because substantial storage and computer memory are required for clustering analysis if implemented with a personal computer (PC).

To improve the computational efficiency and resolve memory limitation problem, principal component analysis (PCA) was first used to narrow down the whole brain voxels before APC clustering analyses were performed for the remaining voxels (Zhang et al., 2011). And we name this updated scheme as PCA-APC. The fMRI datasets can be processed by PC using PCA-APC when the identified voxels by PCA are involved in the computation. However, when the number of data points is significantly increased, the memory insufficiency problem will appear again and in the end the PCA-APC scheme would fail to process the large datasets from the imaging volumes. In addition, APC is not able to achieve an automatic acquisition of the optimal cluster numbers, which will affect the accuracy and reliability of the established brain networks. In order to resolve these issues, a novel data-driven clustering method, namely SOM-SAPC that combines SOM and supervised affinity propagation clustering (SAPC), was proposed to directly perform pattern clustering of complex functional networks involved in MI and ME. And the developed method has the capability to overcome the memory limitation problem when implemented on a PC.

## Materials and methods

### Developed SOM-SAPC for brain network analysis

To reduce the computation cost involved in the brain network analysis, SOM was first implemented for the analysis of fMRI data. Then the generated experimental data were further processed using SAPC to identify brain activation patterns. In this study, SOM adopts a correlation distance metric between the input vector and weight vectors to spot the winning neuron (Chuang et al., 1999; Ngan and Hu, 1999; Ngan et al., 2002; Peltier et al., 2003; Hausfeld et al., 2014), which is different from previous strategy that utilizes the Euclidean distance (Kohonen, 1982, 1990, 1995; Chuang et al., 1999; Haykin, 1999; Ngan and Hu, 1999; Ngan et al., 2002; Peltier et al., 2003; Hausfeld et al., 2014). When SOM is implemented, each generated exemplar will have one corresponding time course. Then further operations can be performed to find the correlation coefficient (CC) between the time course of each voxel of brain slices and the time courses of all exemplars. The biggest CC will occur when the voxel and its corresponding exemplar are of the same cluster.

To ensure the exemplar's time courses can sufficiently retain brain image information (Peltier et al., 2003; Liao et al., 2008), in this study the number of exemplars, 100 was adopted for Kohonen's SOM analysis of fMRI data. If one exemplar represents one cluster, 100 clusters would be involved for clustering analysis, which might bring difficulties in interpreting fMRI data. Therefore, improved SOM-based fMRI data analyses have resorted to further clustering based on the 100 clusters acquired so as to ascertain patterns of brain functions (Chuang et al., 1999; Ngan and Hu, 1999; Ngan et al., 2002; Peltier et al., 2003; Hausfeld et al., 2014).

Following SOM estimation, SAPC is further conducted for the intensity-normalized time courses of the 100 analysis exemplars. Additionally, because only these exemplars' time courses are subjected to SAPC analysis, the new scheme is able to significantly decrease the number of calculations that may otherwise be involved in clustering analysis. “Further clustering” (Peltier et al., 2003) usually necessitates a prior designated number of clusters. It is noted APC is able to generate the optimal set of exemplars and associated clusters (Zhang et al., 2011). However, only SAPC features an automatic acquisition of cluster numbers via supervising the index of clusters and optimizing the input preferences whereas APC is incapable of such automation.

When the general APC is utilized to analyze the data points, the similarity between a pair of normalized vectors *y*_*i*_ and *y*_*j*_ is defined by $s\left(i,j\right)=-∥y_{i}-y_{j}{∥}^{2},i\neq j$. Meanwhile, self-similarity *s*(*i*, *i*) is considered as an input preference (*p*) (Frey and Dueck, 2007; Guan et al., 2011). In APC, the value of *p* as a constant can influence the clustering quality and the number of clusters. However, it is very difficult to specify beforehand the exact *p* because that case is most suitable for a given problem. To search for an optimized *p*, the improved method SAPC has to be employed, which is able to directly supervise the Silhouette index of clusters (Kaufman and Rousseeuw, 1990; Zhang et al., 2011). The calculation of silhouette values is accomplished via the SILHOUETTE function of MATLAB (silhouette is plotted using the Euclidean distance). In particular, the averaged silhouette values represent the clustering quality, in which a larger value will generally provide a more accurate estimate of the clustering quality.

The Golden-section search (Antoniou and Lu, 2007) of the input preferences *p* is implemented in this study to generate an optimal set of clusters. In the Golden-section search, iterations are run until the desired accuracy for the maximum value of the mean silhouette is achieved (Antoniou and Lu, 2007). As a result, a better clustering quality is realized and the cluster number is also identified. Finally, the voxels for each slice of the brain corresponding to the 100 exemplars are clustered based on the clustering results of the 100 exemplars. The index of brain activity is defined as λ_*i*_ = *max*(*l*(*i*)) − *l*(*i*) (Zhang et al., 2011), in which *l*(*i*) determines the Euclidean distance of the *i*th point with its associated cluster center, whereas the maximum *max*(*l*(*i*)) is subject to the same cluster.

### Generation of simulated data

**Figure 1:**
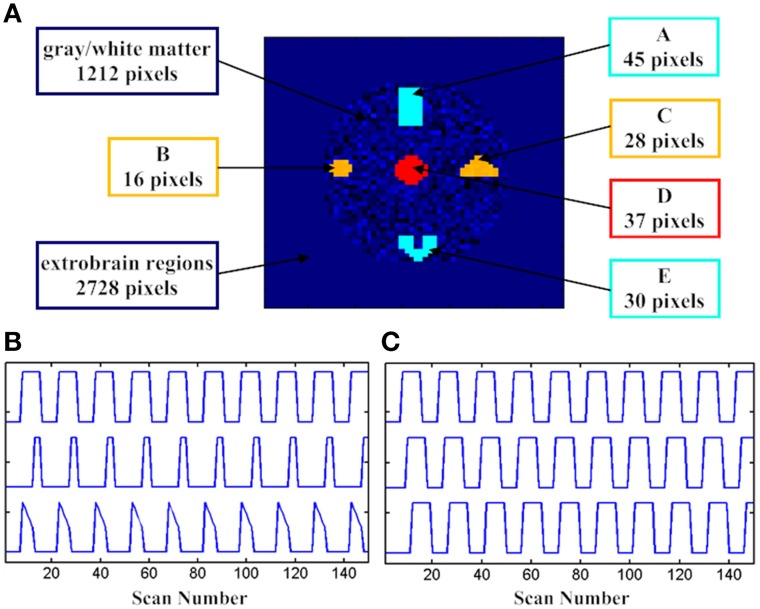
**The spatial and temporal activation patterns for the fMRI simulation tests. (A)** The distribution of assumed active voxels; **(B)** three assumed stimulation patterns in DS1, and **(C)** three assumed stimulation patterns with three delayed versions in DS2.

Simulation tests based on the datasets generated with different hemodynamic responses were first conducted to examine the performance of the developed SOM-SAPC. For the test geometry, a composite image with 4096 voxels was generated on an axial brain. The simulated fMRI image consists of 5 active areas with 156 voxels. In addition, a time invariant margin that has 2728 voxels is added in the test geometry whereas a stochastic variant texture is specified for gray/white matter and ventricles that have 1212 voxels. The 5 brain areas won't have identical sizes and shapes as shown in Figure 1A.

The supposed box-car-like signals, DS1, DS2, and DS3, which are present in the 5 brain activity regions, are utilized here to define the brain activation patterns. DS1 is used to describe the change of the hemodynamic responses to different stimuli patterns. In addition, as displayed in Figure 1B, subregions “A_E” (“A” and “E”), “B_C” (“B” and “C”), and “D” have different temporal signals. The spatial pattern for “A_E” was remote while its temporal pattern was accordant. Both the spatial and temporal patterns were combined together to generate a homogeneous active source for “A_E” and we did the same thing for “B_C” and “D”. Gaussian noise was also added to the time course of each voxel from the subregions with signal-to-noise ratio (SNR) equal to 1.0.

DS2 is able to show the timing variability between a stimulation paradigm and an activation delay of the hemodynamic response. The spatial pattern and SNR from DS2 were identical with those from DS1. Three temporal patterns with three delayed formula (delays of 0, 4, or 8 s; Figure 1C) of the “expected” box-car-like timing function are displayed in the first row of Figure 1C.

DS3 consisted of a given signal mixed with Gaussian noise, and the given signal was depicted in the first row of Figure 1B. DS3 was used to demonstrate the SNR variability. Five sources (subregions “A” and “E,” subregions “B” and “C” and subregion “D”) were correlated with the “expected” boxcar-like timing function. The differences in SNRs (1.2, 1.0, 0.8, 0.6, and 0.4) were computed using the integrated clustering approach.

### Experimental paradigm

#### Subjects

Ten right-handed subjects (aged: 19–25 years) were recruited to participate in the fMRI study. All the participants have normal visual acuity. Nobody has reported neurological diseases or mental disorders in the past 10 years. The subjects were assessed by the Edinburgh Handedness Inventory to exclude any possible left-handers. The clinical tests were consented by the subjects and the local Institutional Review Board of the West China Hospital of Sichuan University.

#### Tasks

**Figure 2:**
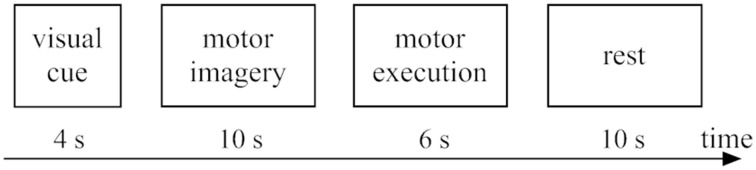
**Experimental tasks within one trial: 4 s for visual cue (sequence informing), 10 s for motor imagery, 6 s for motor execution, and 10 s for rest**.

The experimental design for the present work was the same as our previous fMRI studies (Chen et al., 2009). fMRI tests were performed with 2 conditions including bimanual MI and ME, which covered 10 trials (runs). The stimulus duration for each run was 30 s, as displayed in Figure 2. The stimuli with block design tasks started with sequence informing (4 s), and then followed by MI (10 s), ME (6 s), and resting (10 s). During each run, participants need be familiar with four sequentially pictures. And each sequentially picture is composed of a specific order of finger tapping task. Then, subjects began to perform finger tapping imagery tasks with the same order that was previously informed by the visual stimulus tasks. It should be noted the screen was kept black during 10 s period for MI. Then a new cue was shown on the screen for 6 s, which requested the subject to implement a finger tapping task (ME) with the same content as they had imaged. For our fMRI experimental design, the reason why MI was followed by ME is to make sure the subjects are able to concentrate on the MI task. Before fMRI tests, we would train the participants so that they could perform the experiments very well.

#### Data acquisition

Experimental tests were conducted using a 3.0-T GE Signa scanner (MR Research Center of the West China Hospital of Sichuan University, Chengdu, China). The gradient-recalled echo planar imaging (EPI) sequence settings were as follows: 30 transverse slices, TR = 2000 ms, TE = 30 ms, FOV = 24 cm, acquisition matrix = 64 × 64, slice thickness = 5 mm (without gap), voxel size = 3.75 × 3.75 × 5 mm^3^, and flip angle = 90°. We collected 155 images for the fMRI tests and the data acquisition time was about 310 s.

### Data processing procedure

Experimental data were processed using SPM8 software (http://www.fil.ion.ucl.ac.uk/spm/). In consideration of magnetization equilibrium and for the subjects to be familiar with the procedures (Chen et al., 2009), the first 5 images acquired would be rejected for each run while the additional 150 ones would be kept and calibrated using the methods developed in SPM8. In addition, realignment was adopted to eliminate the head movement. Spatial normalization was also implemented for the images based on the MNI EPI template. Further, fMRI data were smoothed with a FWHM of 8 mm. Ultimately signals captured in the brain areas were processed to validate SOM-SAPC.

## Results

### Simulation tests

#### Compared with PCA

**Figure 3:**
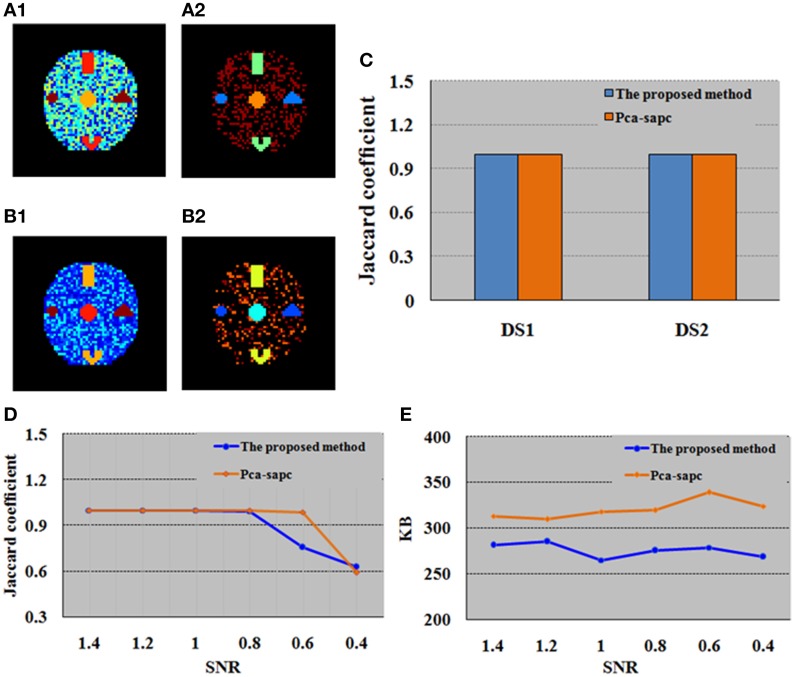
**Comparison between the proposed SOM-SAPC and PCA-SAPC for the simulation tests. (A1)** Clustering results (17 clusters) computed by SOM-SAPC for dataset DS1. **(A2)** Clustering results (12 clusters) computed by SOM-SAPC for dataset DS2. **(B1)** Clustering results (4 clusters) computed by PCA-SAPC for DS1. **(B2)** Clustering results (5 clusters) computed by PCA-SAPC for DS2. **(C)** JCs of DS1 and DS2 calculated by SOM-SAPC and PCA-SAPC, respectively. **(D,E)** are the findings of DS3 with different SNRs. **(D)** are results of JCs while **(E)** describes the maximum memory used by SOM-SAPC and PCA-SAPC, respectively.

To compare with SOM, PCA is first adopted to generate the primary image before the SAPC analysis can be further implemented for the primary image. In PCA, the selected time courses and their numbers are determined by principal components and the selected threshold value (Zhang et al., 2011). In this study, to ensure the chosen principal components retain enough primitive information, the cumulative sum of the variances is set as 90%. For PCA (Zhang et al., 2011), out-of-memory won't occur when performed with a PC (Intel(R) Core (TM) i5 CPU @ 2.67 GHz, RAM 4 GB) for this simulation case. We found that compared with PCA, SOM enables a more efficient clustering analysis and the computational cost based on SOM method was also much lower, as displayed in Table 1. The disparity between the results after SOM and PCA analysis was due to the fact that the quantity of data left over for SAPC analysis after SOM-based processing is far less than that after the PCA-based acquisition of the primary image (Table 1). The identified brain activity regions and the maximum memory utilized by SOM-SAPC were compared to that from PCA-SAPC when they were implemented to process datasets DS1 and DS2 (see Figures 3A1–C) for the reconstructed images and Table 1 for the used maximum PC memory (MB). It was observed from Figure 3 that all the five brain activity regions can be clearly identified by both SOM-SAPC and PCA-SAPC, and the Jaccard coefficient (*JC*) values of both SOM-SAPC and PCA-SAPC are equal to 1. However, it was also found from Table 1 that less memory was required for the SOM-SAPC. In addition, the *JC* values and maximum memory required for different SNRs were also computed and compared between SOM-SAPC and PCA-SAPC for the analysis of datasets DS3 (see Figures 3D,E). We can see that under SNRs ≥ 0.8, the *JC*-values of SOM-SAPC is almost equal to that from PCA-SAPC. However, when SNR = 0.6, the *JC*-value of SOM-SAPC is smaller when compared to that of PCA-SAPC. Figure 3E also showed that SOM-SAPC has smaller memory requirements for all the cases with various SNRs.

**Table 1:** **Discrepancies between PCA and SOM-based analysis via Matlab**.

**Dataset**	**Method**	**Number of time courses left over for SAPC**	**Computation time of SAPC (seconds)**	**Maximum memory utilized (MB)**	
DS1	SOM	100	30.7206	SOM-SAPC	274.248
	PCA	519	146.0798	PCA-SAPC	324.508
DS2	SOM	100	30.9426	SOM-SAPC	276.576
	PCA	432	108.2746	PCA-SAPC	319.140

#### Compared with other clustering analyses

**Figure 4:**
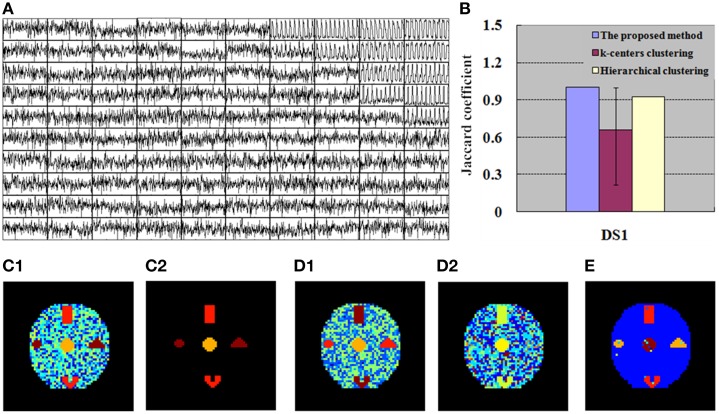
**Finding of DS1 for the simulation tests. (A)** 100 exemplar time courses generated by SOM algorithm. **(B)** *JC* calculated by the integrated method (SOM-SAPC), k-centers clustering and hierarchical clustering, respectively. The blue value is related to one run of SOM-SAPC, the purple value corresponds to 50 runs of k-centers clustering, and the yellow value is correlated with a single run of hierarchical clustering. **(C1)** Clustering results (17 clusters) computed by SOM-SAPC. **(C2)** Reconstructed image with SOM-SAPC when the number of neighborhood voxels in a cluster > 5. **(D1)** The clustering results generated by k-centers clustering with the maximum *JC*-values (for 50 repetitions of the methods), and **(D2)** the minimum *JC*-values. **(E)** The clustering result produced by hierarchical clustering.

**Figure 5:**
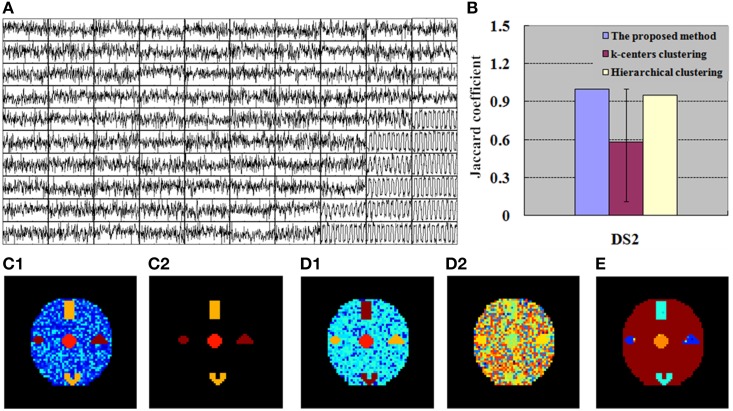
**Finding of DS2 for the simulation tests. (A)** 100 exemplar time courses generated by SOM algorithm. **(B)** *JC* calculated by the integrated method (SOM-SAPC), k-centers clustering and hierarchical clustering, respectively. The blue value is related to one run of SOM-SAPC, the purple value corresponds to 50 runs of k-centers clustering, and the yellow value is correlated with a single run of hierarchical clustering. **(C1)** Clustering results (12 clusters) computed by SOM-SAPC, **(C2)** Reconstructed image with SOM-SAPC when the number of neighborhood voxels in a cluster > 5. **(D1)** The clustering results generated by k-centers clustering with the maximum *JC*-values (for 50 repetitions of the methods), and **(D2)** the minimum *JC*-values. **(E)** The clustering result produced by hierarchical clustering.

**Figure 6:**
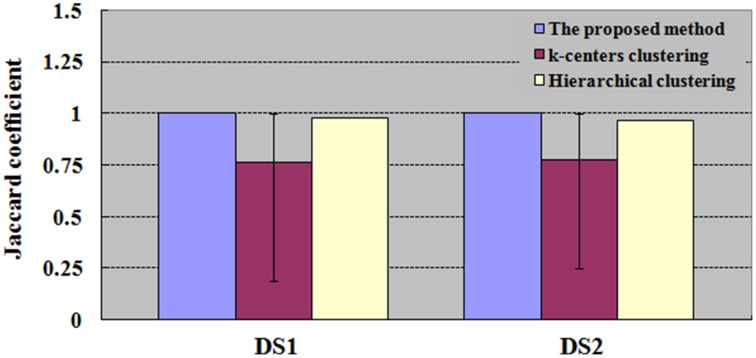
**Results of** ***JC*** **for datasets DS1 and DS2**. The values of k-centers clustering and hierarchical clustering are maximum when the numbers of clusters are ranged from 6 to 100. For each number of clusters, there are 50 runs of k-centers clustering and a single run of hierarchical clustering.

**Figure 7:**
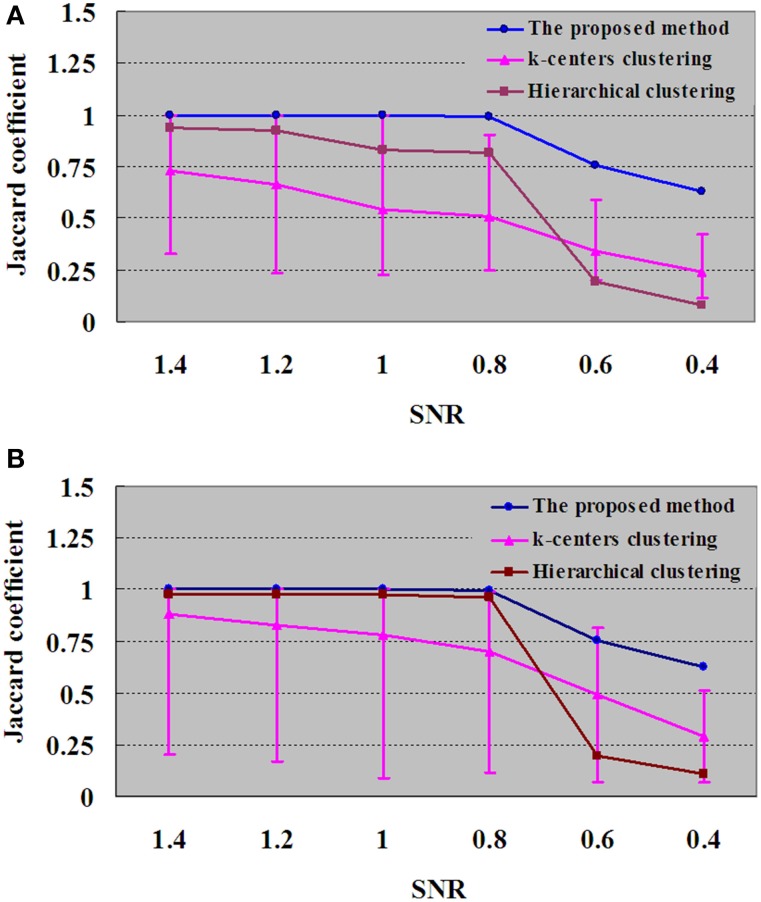
**Results of** ***JC*** **for dataset DS3 under different SNRs. (A)** For each SNR, the *JC* is generated by one run of the proposed method, which will produce a definite clustering result and number of clustering. With the same number of clusters as in the proposed method, the *JC* is generated by 50 runs of k-centers clustering and a single run of hierarchical clustering vs. the SNR. **(B)** For each SNR, the values of k-centers clustering and hierarchical clustering are maximum when the numbers of clusters are ranged from 6 to 100. Each number of clustering is followed by 50 runs of k–centers clustering and a single run of hierarchical clustering.

Simulated data were analyzed using different clustering methods to validate and show the advantages of the developed SOM-SAPC. The image analysis results are provided in Figures 4–7, in which each color represents a unique cluster for (c1), (c2), (d1), (d2), and (e), respectively. The *JC* is employed to examine the performance of the cluster (Anderberg, 1973; Baumgartner et al., 1998; Dimitriadou et al., 2004) and *JC* is equal to *a*/(*a*+*b*+*c*). It is noted here *a, b*, and *c* represents the number of true-positive findings (TP), the number of false-negative findings (FN), and number of false-positive findings (FP), respectively.

Figures 4A, 5A show the exemplar matrix (100 nodes) analysis results calculated from SOM. We found that SOM is capable of identifying the winner nodes. In particular, the exemplar time course was also generated for DS1 and DS2 based on SOM.

Figures 4C1–E, 5C1–E display the clustering results produced from SOM-SAPC, *k*-centers clustering (MacQueen, 1967) and hierarchical clustering (HC) analysis in DS1 and DS2, respectively (the distance measured between two data points is the Euclidean distance, and the hierarchical cluster tree is calculated using the shortest distance) (Goutte et al., 1999; Chen et al., 2006; Liao et al., 2008; Ren et al., 2014). As plotted in Figure 4, the hemodynamic responses to different stimulus patterns are clearly identified for DS1. For example, Figure 4C1 displays the analysis results calculated by SOM-SAPC, which generates 17 clusters. Activated subregions “A_E,” “B_C” and “D” were also reorganized to generate three specific clusters. Figure 4C2 plots the imaging results reconstructed when the number of neighborhood voxels within a cluster is over five with SOM-SAPC. With the same number of clusters as in SOM-SAPC, the results from the *k*-centers clustering analysis with the max and min of the 50 *JC*-values [for the 50 repetitions of the method, because *k*-centers clustering often needs to rerun many times with different initializations in an attempt to find a good solution (Frey and Dueck, 2007)] are plotted in Figures 4D1,D2, respectively while the clustering result of the HC is given in Figure 4E. Likewise, the results of DS2, illustrating timing variability between the stimulation paradigm and hemodynamic response (activation delay) with the same number of clusters as that of the proposed method, are provided in Figure 5.

We then implemented *JC* analysis to provide a better comparison for the clustering capabilities among the different methods (shown in Figures 4B, 5B). It is noted from Figures 4B, 5B that the blue column represents the *JC* in the proposed method, the purple column is the *JC* of k-centers clustering, and the yellow column is the *JC* calculated by the HC method. We found from Figures 4, 5 that SOM-SAPC is able to clearly identify the number of clusters. With the same number of clusters, the computed results using k-centers clustering and HC were also obtained. In addition Figures 4B, 5B show the *JC* performance parameters. We observed from Figures 4, 5 that in both SOM-SAPC and HC analyses, each column *JC*-value is generated by a single run, while in k-center clustering, each column *JC*-value is generated by 50 runs. The large *JC*-values are obtained by SOM-SAPC, and pixels in the activated subregions “A_E,” “B_C” and “D” are correctly grouped into three different clusters.

With the same number of clusters as in the integrated method (SOM-SAPC), Figures 4B, 5B plot the *JC* performance. Yet for k-centers clustering and HC, the same number of clusters as in the integrated method is not necessarily the most suitable. Therefore, in Figure 6, the number of clusters for both k-centers clustering and HC is adopted from 6 all the way to 100 for DS1 and DS2. Each number of clusters corresponds to 50 runs of k-centers clustering and a single run of HC. The numbers of clusters, ranging from 6 to 100, have corresponding *JC*s. The maximum *JC* for HC is identified and adopted. For k-centers clustering, each corresponding number of clusters has to run 50 times, and there are consequently 50 *JC*s, whose mean is then calculated and acquired. The number of clusters ultimately gives rise to 95 mean values. We then adopt the number of clusters that corresponds with the biggest mean value, which gives a graphic representation of the mean, maximum and minimum *JC* in relation to this number of cluster under k-centers clustering.

To test the performance of the algorithm with decreased SNR, the SNRs of DS3 were added, ranging from 1.4 to 0.4. Figure 7 displays the *JC* curves obtained from the integrated method, *k*-centers clustering and HC with different SNRs. *JC* analysis is implemented to evaluate the performance of the proposed method and to compare with k-centers clustering and HC methods, and the results are given in Figure 7, in which the blue curve demonstrated that the performances turns to the downside when SNRs were varied from 0.8 to 0.4. In Figure 7A, for each SNR the *JC* is generated by one run of the proposed method, which will produce a definite clustering result and number of clusters. With the same number of clusters as in the proposed method, the *JC* is generated by 50 runs of k–centers clustering and a single run of HC vs. the SNR. In addition, in Figure 7B, for each SNR the number of clusters for k-centers clustering and HC are ranged from 6 to 100, and the values of k-centers clustering and HC are found to be maximum in the same range. And each number of clusters would give rise to 50 runs of k-center clustering and a single run of hierarchical clustering.

### fMRI experimental data test

**Figure 8:**
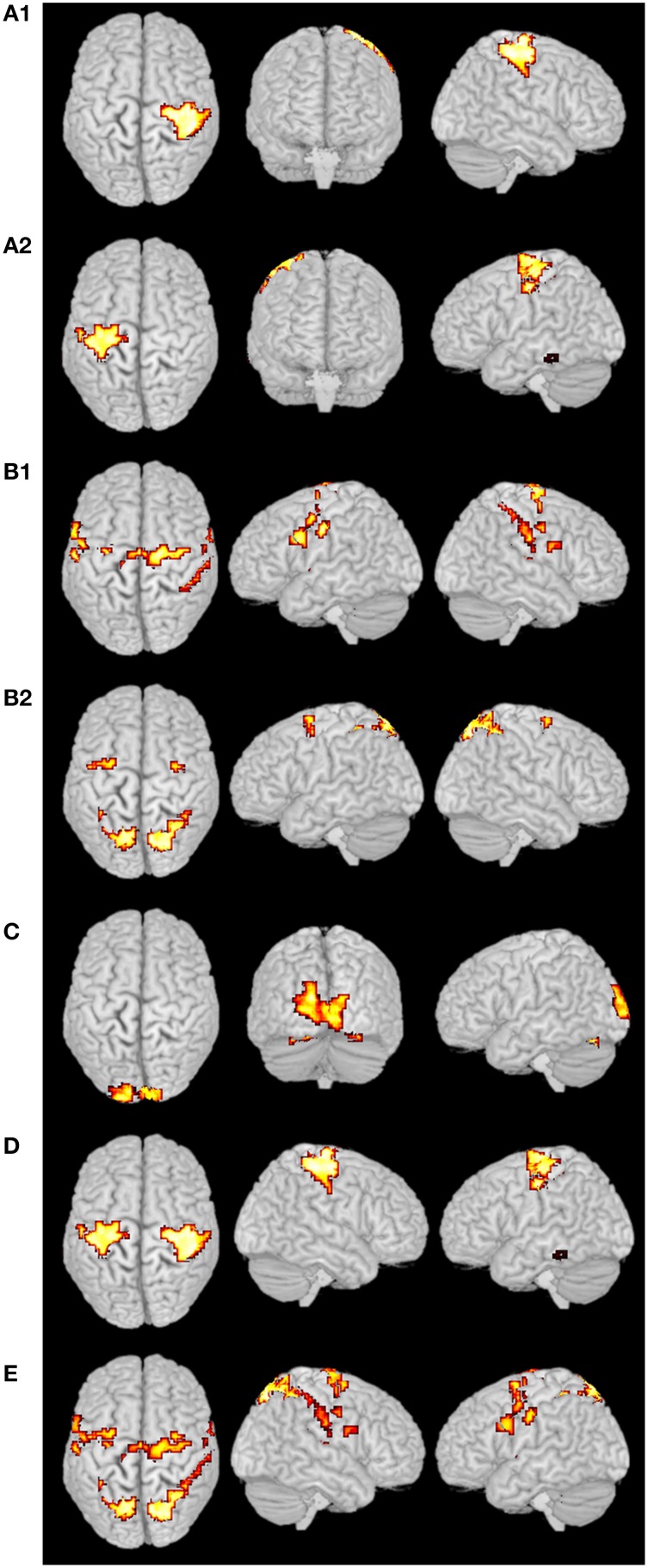
**Clustering results from real fMRI data**. The clusters **(A1)**,**(A2,D)** were the ME networks, and the cluster **(D)** was the integrated clusters from **(A1)** and **(A2)**. The clusters **(B1,B2,E)** were the MI networks, and the cluster **(E)** was the integrated clusters from **(B1)** and **(B2)**. The cluster **(C)** was the primary visual network.

**Figure 9:**
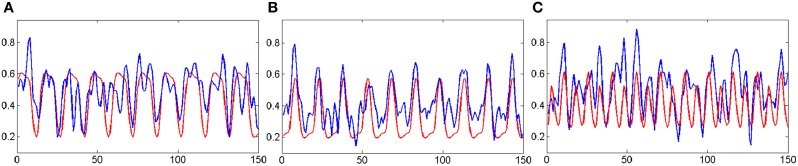
**The correlations between the mean time courses of the activation clusters and the time courses of stimulation patterns of fMRI experiment**. The blue curves represent the mean time courses of the activation clusters while the red curves represent that of the stimulation patterns. The horizontal axis represents image number; the vertical axis represents signal intensity. **(A)** is corresponding to the MI networks in Figure 8E; **(B)** is corresponding to the ME networks in Figure 8D; **(C)** is corresponding to the primary visual networks in Figure 8C.

**Figure 10:**
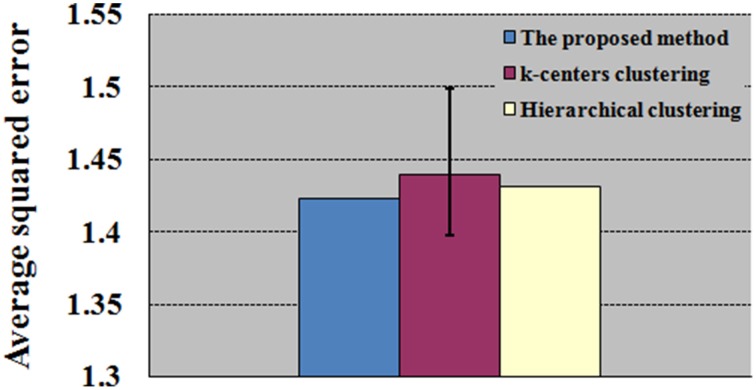
**Comparison between the proposed SOM-SAPC with other clustering approaches for the construction of ME and MI functional networks**. The average squared errors were achieved by a single run of SOM-SAPC, 100 runs of k-centers clustering and a single run of hierarchical clustering.

For the fMRI experiments in this study, subjects were presented with visual stimuli, bimanual MI and ME. These tasks had different durations and were performed sequentially. The primary clustering of the real fMRI data was completed by SOM, which produced relatively less amount of data than by PCA. Then we adopted the developed SOM-SAPC, which were able to reveal the new neural findings and identify the brain networks for both MI and ME using fMRI data. In particular, Figure 8 shows the five clusters of activated cortical networks with the different stimulation patterns while Figure 9 plots the calculated correlation coefficients between the mean time courses of the activation clusters and the different stimulation patterns of tasks in Figures 8C–E. Importantly, the time courses inside a specific cluster were found to be strongly correlated with a certain stimulus pattern. For example, a high correlation coefficient (the coefficient value 0.817 is shown on the third row of Table 2) was revealed (shown in Figure 9B and Table 2) between the mean time courses of components in the clusters (Figures 8A1,A2) and the convoluted experiment design of the ME task. These findings suggested that the clusters were responsible for ME network (shown in Figure 8D, clusters (a1) and (a2) integrated). Moreover, the time courses of the clusters in Figures 8B1,B2 were seen to be highly related to the convoluted stimulation pattern of the MI task (shown in Figure 9A and the coefficient value 0.622 is shown in the second row of Table 2), which indicated that these clusters should be related to MI network (shown in Figure 8E, clusters (b1) and (b2) integrated). Furthermore, the time course of the cluster in Figure 8C was found to be correlated with the convoluted stimulation pattern of the visual cue (shown in Figure 9C and Table 2). Finally, we observed that the spatial locations of the clusters were consistent with those captured by our previous studies (Wang et al., 2010) for the same datasets. In addition, it is very difficult to use these schemes (HC and k-centers clustering) to directly conduct pattern clustering due to the large datasets involved in the analysis of ME and MI functional networks. As such, only part of the datasets from the six slices of the whole-brain are adopted for computation. The comparisons among the proposed SOM-SAPC scheme with other clustering approaches were also performed using the average squared error between the data points and the center of the identified cluster (see Figure 10). We can see from Figure 10 (the number of clusters is 66) that the error calculated from the SOM-SAPC was lower than that from the hierarchical clustering and the mean with 100 runs of k-centers clustering.

**Table 2:** **The correlation coefficients calculated between the mean time courses of the activation clusters and the time courses of stimulation patterns of fMRI**.

**Cluster**	**Correlation coefficient**	**Responding task**
Figure 8E	0.622	Motor imagery
Figure 8D	0.817	motor execution
Figure 8C	0.513	Visual cue

## Discussion

Recently identifying brain networks when brain is activated with multitask has attracted particular attention in the neurocognitive fields (Wang et al., 2010). For example, MI and ME have been extensively investigated using a number of multitask tests with fMRI measurements (Hanakawa et al., 2003, 2008; Hu et al., 2005; Wang et al., 2010). To classify the cerebral functions evoked in experiments, clustering methods were generally implemented to identify brain functional activation patterns (Chuang et al., 1999; Goutte et al., 1999; Ngan and Hu, 1999; Fadili et al., 2000; Mezer et al., 2009). However, few studies based on pattern clustering methods have been conducted using fMRI data for MI and ME network analysis. The PCA-SAPC proposed previously by our group (Zhang et al., 2011) also failed to effectively process the large datasets in order to classify and generate the ME and MI functional networks. In this study, we presented a multi-voxel clustering method, known briefly as SOM-SAPC, by integrating improved SOM and SAPC. This newly developed method is proposed to construct the cerebral networks for MI and ME, enabling the recognition of different signal patterns of brain activation by fMRI data with reduced computational cost. Our results suggested that brain networks and associated brain activation patterns were strongly correlated with different conditions of multitask experiments.

### Discussion on conventional clustering methods

Several data-driven based clustering methods have been utilized to reveal brain activity regions with different signal patterns, (Barth et al., 1997; Baumgartner et al., 1998, 2000; Baune et al., 1999; Chuang et al., 1999; Filzmoser et al., 1999; Moser et al., 1999; Dimitriadou et al., 2004; Mezer et al., 2009), which includes *k*-center analysis (MacQueen, 1967) and hierarchical clustering (HC) analysis tools (Goutte et al., 1999; Chen et al., 2006; Liao et al., 2008; Ren et al., 2014). However, compared with SOM-SAPC, *k*-center analysis cannot resolve the problems like local minima and initialization when implemented with repeated runs. Further, unlike SOM-SAPC, stopping criteria has to be pre-determined for HC. Importantly, the data point pattern for HC can only be classified to a pre-denoted cluster, but not the other clusters (Jain and Dubes, 1988; Frigui and Krishnapuram, 1999; Geva, 1999; Berkhin, 2006).

### Discussion on Kohonen's SOM and PCA

Kohonen's SOM is considered an ingenious neural network which uses a simple geometric computation to replace the more detailed properties of the Hebb-like rule and lateral interactions (Haykin, 1999). For example, SOM has the disadvantages of using the Euclidean distance metric for fMRI signal analysis and for identifying the matched nodes. Compared with the correlation distance metric, Euclidean distance metric is more vulnerable to the baseline level. However, for MI and ME data processing, SOM is still very effective in coping with the time course of whole-brain voxels, which can only produce hundreds of exemplar time courses before the later implementation of SAPC analysis. In contrast, although PCA can also be used to decrease the amount of data prior to SAPC clustering analysis, its mitigating effects on MI and ME data are far from satisfying, because it routinely fails to contribute to a smooth pattern clustering analysis when implemented with SAPC on PCs.

### Discussion on SAPC

SAPC is used to cluster the preliminary results generated by improved SOM, enabling a more complete and in-depth analysis of brain function. In SAPC, the Silhouette index is adopted to identifying the best clustering solution. Among many validity indices of clustering results, the Silhouette index performs very well in terms of calculating the number of clusters with clear cluster structures, which can also be used to judge the quality of the clustering result (Kaufman and Rousseeuw, 1990; Berkhin, 2006). In one-dimensional optimization of preference *p* under SAPC, we have adopted, unlike previously work (Zhang et al., 2011), the Golden-section search, so as to make sure of the accuracy of *p*-value.

### Discussion on the integration of methods

Compared with single only APC method, the developed SOM-SAPC approach is immune to possible memory resource insufficiency in analyzing large fMRI datasets, and able to significantly improve computation efficiency. The results in Figures 4B, 5B, 6, 7 show that the SOM-SAPC has better performance compared to those from *k*-centers clustering and HC in some respects. For example, the initial guesses of exemplars is essential to *k*-center clustering method, in which a bunch of runs with different initial guesses are needed to get more accurate results based on this method (Frey and Dueck, 2007). In contrast, this is not the case for the integrated SOM-SAPC method since we can get better results by supervising the Silhouette index. As such, SOM-SAPC is different with the *k*-centers clustering because it is independent of the initialization of the cluster centers and further provides the optimal set of clusters for a given dataset.

### The integration of functional networks

Figure 8 shows the overlapped activated regions in ME and MI during the associated tasks, which reveals clusters that represent the activation of different functional networks in task-related states. It should be pointed out that the findings in Figure 8 were in good agreement with the previously reported results (Lotze et al., 1999; Hanakawa et al., 2003; Lacourse et al., 2005; Wang et al., 2010). In our experiment, subjects were in a variety of task states, and each task state has its unique functional networks. Because of the functional asymmetry of cortical activity in motor areas during sequential finger movements (White et al., 1994; Amunts et al., 1996, 1997a,b, 2000; Dassonville et al., 1997; Volkmann et al., 1998; Yuan and Ye, 2013; Pool et al., 2014), the left and right motor areas show the differences in BOLD signal magnitude changes. Basically, activated regions responding to ME tasks were classified into two clusters (i.e., Figures 8A1,A2). For example, the activated regions in Figures 8A1,A2) could be denoted as the same functional network (shown in Figure 8D) since the averaged time courses of components within these clusters were found to have strong correlation with the stimulation pattern of the ME task as compared to the corrections from other tasks (see Figure 9B and Table 2). The activated regions in Figures 8B1,B2 were able to be classified into one MI networks (shown in Figure 8E) because we found the mean time courses from components inside these specific clusters (Figures 8B1,B2) were shown to be strongly correlated with the convoluted experiment design of the responding tasks (Figure 9A and Table 2). In addition, the clusters in Figures 8B1,B2 consist of the same brain activity areas as previous findings (Hanakawa et al., 2003), which indicated brain networks integrated among the activated regions were essential to complete the MI task in our experiment.

## Conclusion

In this study, we proposed a novel method, namely SOM-SAPC, which is able to integrate improved SOM and SAPC for the clustering of brain activity patterns of multitasks related to MI and ME. The developed method is able to handle large datasets using PC and is very efficient for brain connectivity analysis while the clustering method based on the combination of PCA and APC could hardly cope with such large datasets for construction of brain networks. Our fMRI findings validated that SOM-SAPC could reveal patterns of brain activity in response to multitasks. In particular, several unique brain networks that involved in performing the multitasks related to MI and ME were identified, which include parts of the visual, ME and MI functional networks. We suggested that the developed scheme is very sensitive to fMRI measurements and can effectively capture brain activity patterns and networks using fMRI data.

### Conflict of interest statement

The authors declare that the research was conducted in the absence of any commercial or financial relationships that could be construed as a potential conflict of interest.
